# P16^INK4a^ expression in patients with penile cancer

**DOI:** 10.1371/journal.pone.0205350

**Published:** 2018-10-12

**Authors:** Vicenilma de Andrade Martins, Jaqueline Diniz Pinho, Antonio Augusto Lima Teixeira Júnior, Leudivan Ribeiro Nogueira, Fábio França Silva, Victor Eduardo Maulen, André Salim Khayat, José de Ribamar Rodrigues Calixto, Herikson Araújo Costa, Leandra Naira Zambelli Ramalho, Gyl Eanes Barros Silva

**Affiliations:** 1 Postgraduate Program in Adult and Child Health (PPGSAC), Federal University of Maranhão (UFMA), São Luís, Brazil; 2 Postgraduate Program in Genetics and Molecular Biology (PPGBM), Federal University of Pará (UFPA), Belém, Brazil; 3 Postgraduate Program in Adult Health (PPGSAD), Federal University of Maranhão (UFMA), São Luís, Brazil; 4 Maranhense Institute of Oncology Aldenora Bello (IMOAB), São Luís, Brazil; 5 Northeast Network of Biotechnology Program (RENORBIO), State University of Ceará (UECE), Ceará, Brazil; 6 Presidente Dutra University Hospital (HUPD), São Luís, Brazil; 7 Department of Medicine II, Federal University of Maranhão (UFMA), São Luís, Brazil; 8 Department of Physical Education, Federal University of Maranhão (UFMA), Pinheiro, Brazil; 9 Department of Pathology, Ribeirão Preto Medical of School, University of São Paulo (USP), Ribeirao Preto, Brazil; Istituto Nazionale Tumori IRCCS Fondazione Pascale, ITALY

## Abstract

**Background:**

Infection with human papillomavirus (HPV) is reported to be present in 30–50% of penile cancer cases. The immunohistochemical test for p16^INK4a^ is used as an indicator of the presence of HPV and as a prognostic marker for squamous cell carcinomas in various sites. However, the role of this marker in penile carcinoma has not yet been completely elucidated. The aim of this study was to analyze whether the expression of p16^INK4a^ is associated with the presence of HPV, histological parameters, and survival in penile cancer.

**Methods:**

A study was conducted from 2014 to 2016 that included 55 patients with penile carcinoma. HPV DNA was detected through PCR using fresh tumor tissue, and immunohistochemistry was performed for analysis of p16^INK4a^ protein using paraffin-embedded tissue. Evaluation of histological parameters was performed following complete embedding of the tumor tissue in paraffin.

**Results:**

HPV DNA (low-risk and high-risk genotypes) was found in 49 (89.1%) cases, and 46/49 (93.9%) showed high-oncogenic risk HPV (HR-HPV). Of the 22 cases positive for p16^INK4a^, HR-HPV DNA was present in 21 (95.5%) (p = 0.032). Regarding histological parameters, p16^INK4a^ and HR-HPV were significantly associated only with tumor subtype (p = 0.036 and p = 0.032, respectively); all carcinomas with basaloid characteristics were positive for p16^INK4a^. Although HPV+ patients had a higher disease-free survival (p <0.001), p16^INK4a^ expression was not associated with patient survival.

**Conclusions:**

Our study, using fresh tissue samples, showed the highest incidence of HPV compared to that observed in the literature. Expression of the p16^INK4a^ protein was significantly associated with the presence of HR-HPV and this expression may serve as a marker for the presence of the virus. The p16^INK4a^ protein was not associated with the histological prognostic parameters, with the exception of tumor subtype, nor with patient survival. In the results, we showed that the objective of the present study was reached.

## Background

Penile cancer (PC) is considered a rare neoplasm in developed countries [1, 2]. However, in developing countries in Asia, Africa, and South America, it is considered a public health problem [3–5]. Unpublished data show that the northeastern region of Brazil, in particular the State of Maranhão, has the highest registered incidence of PC in the world.

Among several risk factors for the development of PC, infection with human papillomavirus (HPV) is one of the most highly associated factors reported in the literature. The incidence of HPV infection in penile carcinoma ranges from 30 to 50% [6–9], particularly for the high-risk oncogenic subtypes [6,10–12]. However, the biomolecular mechanisms underlying the association of HPV with PC are not fully understood; the mechanisms are believed to be similar to those in cervical and vulvar carcinoma, where the E6 and E7 viral proteins bind to and inactivate tumor suppressor gene products such as p53 protein and retinoblastoma protein (pRb), both of which are responsible for negative control of cell proliferation [13, 14]. E7 protein inhibits pRB activity, leaving the E2F transcription factor free and triggering DNA replication, thereby resulting in p16^INK4a^ protein overexpression by negative feedback in infected cells [15, 16].

Immunohistochemical expression of p16^INK4a^ is often used as an alternative marker for the presence of high-oncogenic risk HPV in cervical neoplasias as well as squamous cell carcinoma in other organs [17–19]. This test has also been used to aid in diagnosis of high-grade squamous intraepithelial lesions to predict the progression or regression of low-grade cervical intraepithelial neoplasia and as a marker of better cancer-specific survival (CSC) [20–24]. However, little is known about the association of HPV infection with p16^INK4a^ expression in patients with PC. Thus, the objective of the present study was to analyze if there was an association between the expression of P16^INK4a^ protein and: 1) histological parameters present in tumors that were totally included in paraffin; 2) the presence of HPV in patients with PC; and 3) patient survival.

## Methods

The present study included analysis of 55 cases of penile carcinoma. Diagnosis was confirmed by anatomopathological diagnosis and all patients underwent a surgical procedure at the Presidente Dutra University Hospital or the Aldenora Bello Maranhense Institute of Oncology between 2014 and 2016.

Data were collected from an interview with the patients, analysis of histopathological reports, and review of patient medical records. Samples of paraffin-embedded tumor tissues were selected for immunohistochemistry, and fresh tissue samples were used for the polymerase chain reaction (PCR) analysis.

### Histological identification of HPV and other histological parameters

**Fig 1:**
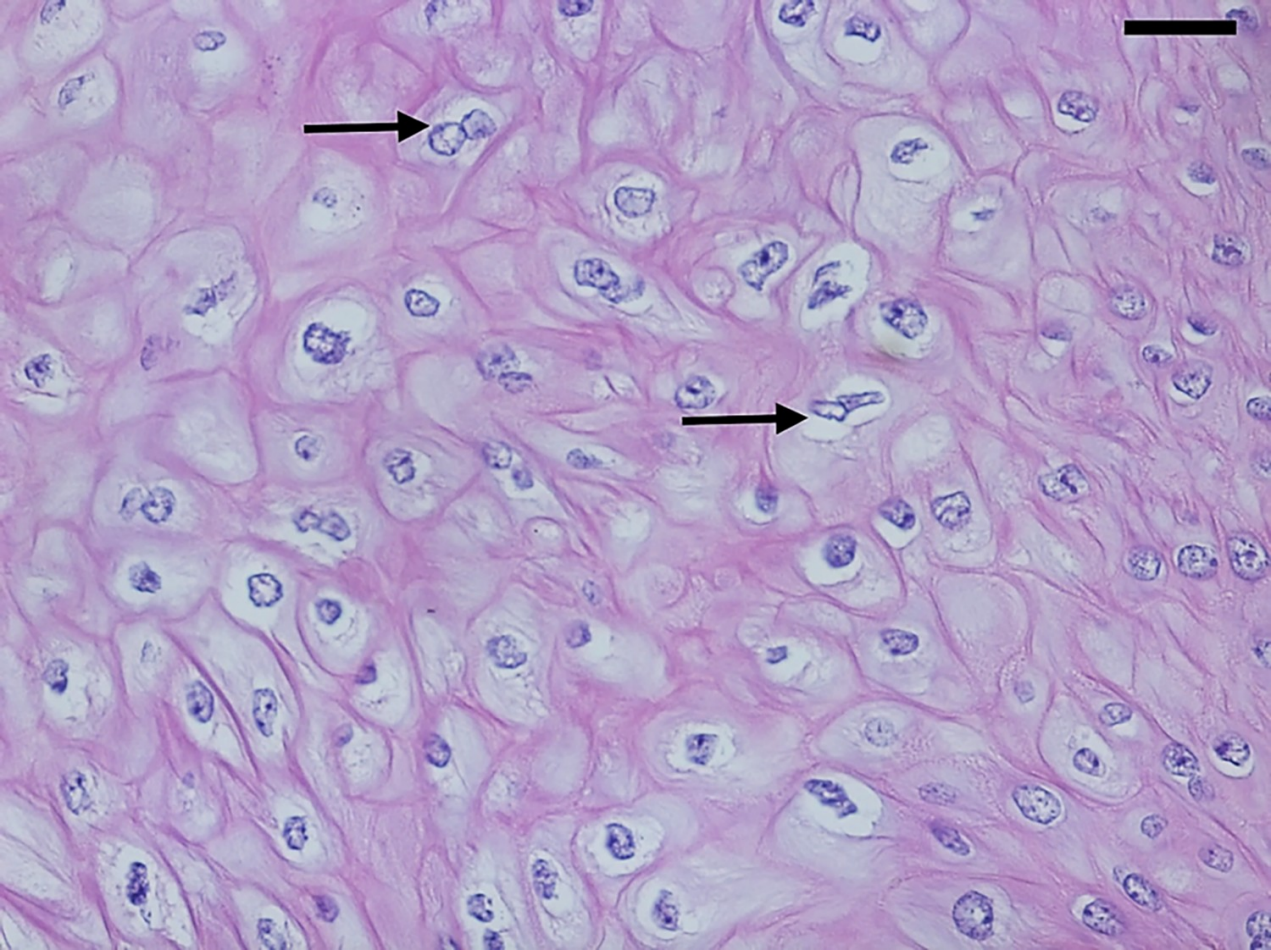
Histopathology of patient's penile tissue, stained with hematoxylin-eosin. Arrow indicates presence of koilocytosis. 1000× magnification.

The histological diagnosis of HPV was defined by the presence of koilocytosis, with three obligatory criteria: a) perinuclear halo, b) nuclear atypia, and c) binucleation (Fig 1). To increase the reliability of the histological parameters, our protocol included the complete penectomy sample (including prepuce), except in cases with more than 50 cassettes. Macroscopic examination and histopathological evaluation were performed by a urologist with 15 years of experience (GEBS), who analyzed all slides and was blinded to the molecular results of HPV screening. The 7^th^ edition of the American Joint Committee on Cancer (AJCC) Cancer Staging Manual was used to classify tumor staging [25].

### HPV molecular analysis

To determine the presence or absence of HPV DNA in the PC samples, DNA extraction with phenol-chloroform was performed according to the protocol of Sambrook et al. (1989) [26]. Subsequently, nested PCR was performed in two stages. In the first stage, a set of generic primers called PGMY09/11, described by Gravitt et al. (2000) [27], were used. These primers produce a 450-bp fragment of the HPV L1 region. In the second stage, the primer GP5+/6+ [28] was used, generating a 170-bp amplicon, which corresponds to the L1 region of the viral capsid. In addition, a pool of HPV 18-positive HeLa cells was used as a control. The amplicons were separated on a 1.5% agarose gel, subjected to a constant voltage of 90 V for 40 min.

HPV genotyping was performed for the samples identified as HPV-positive by the sequencing reaction with the DYEnamic ET Terminator Cycle Sequencing Kit, according to the protocol suggested by the manufacturer (GE Healthcare, Little Chalfont, UK).

### Immunohistochemistry for the expression of p16^INK4a^

**Fig 2:**
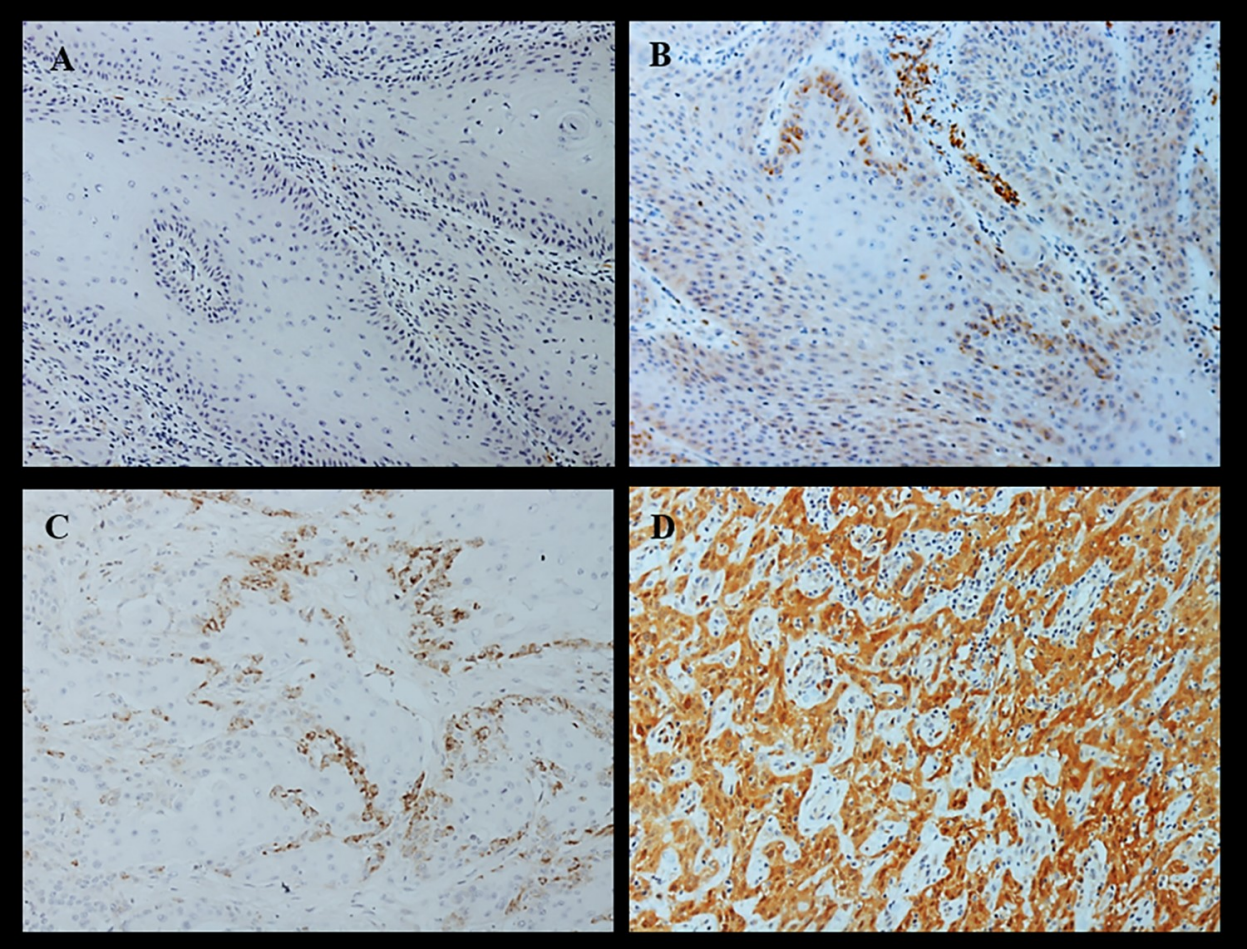
Patterns of p16 expression in penile carcinomas. (A) Pattern 0: complete absence of staining in all neoplastic cells. (B) Pattern 1: irregular and discontinuous individual staining in some of the neoplastic cells. (C) Pattern 2: a more extensive, although discontinuous, staining pattern with small clusters of positive neoplastic cells. (D) Pattern 3: continuous and complete cytoplasmic and nuclear staining in all neoplastic cells. Only pattern 3 was considered positive for p16.

Immunohistochemistry to determine the expression of p16^INK4a^ was performed according to the manufacturer's protocol using a mouse monoclonal primary antibody p16^INK4a^ Clone G175-405 (Zeta Corporation, Arcadia, CA, USA) diluted at a ratio of 1:75 μc. Blade evaluation and image acquisition were performed using a Zeiss optical microscope (AxioCam MRC) and image acquisition software (Zen 2012) (Zeiss AG, Oberkochen, Germany). To define the expression patterns of p16^INK4a^, the classification of Cubilla et al. (2011) was adapted [6], where the superficial portion of the epithelium was excluded and coloring topography (nuclear and cytoplasmic) was used. Accordingly, four p16^INK4a^ expression patterns were found and were categorized as follows: pattern 0, complete absence of staining in all neoplastic cells; pattern 1, irregular and discontinuous individual staining in some of the neoplastic cells; pattern 2, a more extensive, although discontinuous, staining pattern with small clusters of positive neoplastic cells; and pattern 3, continuous and complete cytoplasmic and nuclear staining in all neoplastic cells. Only pattern 3 was considered positive for p16^INK4a^ expression (Fig 2).

### Statistical analysis

Data were tabulated using Excel (Microsoft Office 2013) and analyzed with SPSS V22 (IBM Corp., Armonk, NY, USA). Relative and absolute frequency analyses were performed to verify the distribution of the categorical variables. A Kolmogorov-Smirnov test was used to analyze the normality of the quantitative variables with these variables expressed as mean and standard deviation. The association between HPV status and p16^INK4a^ protein expression was determined by the Chi-square test. Survival analysis was performed using the Kaplan-Meier method to determine disease-free survival and overall survival. The log-rank test was used to compare survival curves. The significance threshold used was p ≤ 0.05.

### Ethical aspects

The study was approved by the Scientific Commission—COMIC—HUUFMA with the opinion no. 2457/2014-60 and by the Research Ethics Committee of the University Hospital/CEP-HUUFMA with approval number: 1.093.435. To meet the regulations for research with human subjects, established by Resolution no. 466/12 of the National Health Council, all duly guided participants provided a signed Free and Informed Consent Form (FICF).

## Results

### Clinicopathological characteristics

We analyzed 55 patients with PC, with a mean age of 61 ± 17.87 years at the date of surgery. Among these patients, 44 (80%) were married or in a stable relationship, 26 (47.3%) were farmers, 50 (90.9%) had a low educational level (illiteracy or elementary school), and 29 (52.7%) were smokers. Phimosis was present in 40 (72.7%) cases.

Among the cases included, 28(50.9%) were of subtypes of cancer associated with the presence of HPV, which include warty, basaloid, or mixed carcinomas with warty or basaloid components.

Pathological cancer staging with high metastatic potential (pT1b to pT3 stage) was observed in 44 cases (80%), and 43 (78.2%) were classified as tumors of low degree of differentiation (Broders degree GI-II). Perineural or lymphatic invasion was present in 19 (34.5%) and 16 (29.1%) cases, respectively. Histological evaluation revealed the presence of koilocytosis in 41 cases (74.5%). A more detailed description of the histological characteristics is presented in Table 1.

**Table 1:** Distribution of histological characteristics and HPV infection status.

Variables		*N*	*%*
**Histological type**	Epidermoid carcinoma	55	100%
**Tumor subtype**	Usual	26	47.3%
	Warty	16	29.1%
	Basaloid	4	7.3%
	Mixed*	9	16.3%
**Tumor size**	≤ 0.5 cm	1	1.9%
	0.6–2 cm	7	12.7%
	2.1–5.0 cm	34	61.8%
	≥ 5.1 cm	13	23.6%
**TNM (AJCC, 8°ed.)**	pTx	3	1.8%
	pT1—pT1a	8	9.1%
	pT1b - pT3	44	80.0%
**Broders degree**	Degree I	9	16.4%
	Degree II	34	61.8%
	Degree III	12	21.8%
**Perineural invasion**	Present	19	34.5%
	Absent	36	65.5%
**Lymphatic invasion**	Present	16	29.1%
	Absent	39	70.9%
**Phimosis**	Present	40	72.3%
	Absent	15	27.3%
**Koilocytosis**	Present	41	74.5%
	Absent	14	25.5%
**Lymphadenectomy**	Present	17	30.9%
	Absent	38	69.1%

N: Absolute frequency

%: Relative frequency

* Mixed: 2 usual and basaloid, 6 usual and warty, 1 usual and verrucous; DNA: deoxyribonucleic acid; HPV: human papilloma virus.

All patients underwent penectomy; 78.2% underwent partial penectomy and 21.8% total penectomy. The average time from onset of symptoms until the search for treatment was 21.3 months, and the follow-up period was 17.3 months. During the follow-up period, five patients died from cancer, eleven had lymph node metastases, 28 remained alive without disease, and four did not undergo follow-up, while seven experienced recurrence. Regarding the type of complementary treatment, two patients were administered chemotherapy and seven received radiotherapy.

### HPV genotyping

HPV DNA was present in 49 cases (89.1%), of which 46 (93.9%) were classified as high-oncogenic risk HPV (HR-HPV) and 3 (6.1%) as low-oncogenic risk HPV (LR-HPV). Among the high-risk subtypes, the most frequent was HPV 16 (69.5%). The remaining HPV subtypes associated with high oncogenic risk were either observed alone or together with other subtypes. The low-risk subtypes included subtype HPV 11, HPV 6 and HPV 30. In addition, two cases of multiple infection with the HPV subtypes 35 and 59 was observed (Table 2).

**Table 2:** HPV identification and genotyping.

		*N*	*%*
**HPV DNA**	HPV-Positive	49	89.1%
	HPV-Negative	6	10.9%
**HPV Genotype**	HR-HPV	46	93.9%
	LR-HPV	3	6.1%
**HR-HPV**	16	26	56.5%
	16/6	1	2.1%
	16/74	2	4.4%
	16/30	1	2.1%
	16/66	1	2.1%
	16/44/74	1	2.1%
	35/59	2	4.4%
	*52, 53,58,(58/51), 59, (30/66),(66/59), (73/74), 74, (11/35/59)	10	22%
	56	2	4.3%
**LR-HPV**	11	1	33.3%
	11/6	1	33.3%
	30	1	33.3%

N: Absolute frequency

%: Relative frequency

*Genotypes occurring only once.

### P16^INK4a^ protein and HPV

The distribution of expression patterns of the p16^INK4a^ protein is shown in Table 3. Among the cases, 33 (60%) were negative for the p16^INK4a^ protein and 22 (40%) were positive. Among the p16^INK4a^ positive cases, 11 (50%) were usual carcinomas and the other 11 (50%) were basaloid, warty, or mixed with warty or basaloid components. HR-HPV DNA was present in 21 (95.5%) of the p16^INK4a^-positive cases. Among the LR-HPV cases, only one was considered p16^INK4a^-positive.

**Table 3:** Distribution of p16^INK4a^ protein expression.

	Expression pattern	*N*	*%*
**P16**	0	5	9.1%
	1	6	10.9%
	2	22	40%
	3	22	40%

Expression pattern of p16^INK4a^: 0—absence of immunostaining; 1—stained, irregular and discontinuous immunostaining; 2—more extensive, discontinuous immunostaining, small agglomerates; 3—continuous and complete cytoplasmic and nuclear staining.

### Association of p16^INK4a^ expression with histological characteristics and high-risk HPV infection

After exclusion of LR-HPV cases from analyses and considering the histopathological characteristics of PC, an association was observed between p16^INK4a^ positivity and tumor subtype (p = 0.036); 100% of basaloid or mixed basaloid carcinomas were p16^INK4a^ positive. When basaloid or mixed basaloid carcinomas were excluded from analyses, no association with p16^INK4a^protein overexpression was found. Other histological characteristics of the tumors were not found to be significantly associated with p16^INK4a^ (Table 4).

**Table 4:** Association between p16^INK4a^ protein, histological characteristics, and HR-HPV infection status.

Characteristics	*P16 +*	*P16 -*	P value*
**Tumor subtype**			
Basaloid	4	0	0.036
Warty	3	12	
Mixed	4	5	
Usual	10	14	
**Tumor size**			
≤ 2 cm	2	6	0.335
> 2 cm	19	25	
**TNM**			
pTx	2	1	0.631
pT1 to pT1a	3	5	
pT1b to pT3	16	25	
**Phimosis**			
Present	15	22	0.971
Absent	6	9	
**Broders degree**			
Degree 1	3	5	0.741
Degree 2	12	20	
Degree 3	6	6	
**Lymphatic invasion**			
Present	3	11	0.091
Absent	18	20	
**Perineural invasion**			
Present	4	12	0.132
Absent	17	19	
**Lymph node metastasis**			
Yes	3	8	0.318
No	18	23	
**HPV DNA**			
HPV +	22	27	0.034
HPV -	0	6	
**HPV Genotype**			
HR-HPV	21	25	0.032
No HPV	0	6	

HPV: Human papillomavirus; HR-HPV: High-oncogenic risk; P16 +: overexpression of p16^INK4a^ protein; p16-: absence of overexpression of p16INK4a protein; DNA: deoxyribonucleic acid; HPV +: presence of HPV DNA; HPV -: absence of HPV DNA

*: Degree of significance of the associations performed by Chi-square test.

Table 4 illustrates the presence of a significant association between p16^INK4a^ and the presence of HPV DNA (p = 0.034). A significant association was also identified between HR-HPV DNA and p16^INK4a^ (p = 0.032), with 21/46 cases of HR-HPV positive for p16^INK4a^.

### Survival analysis regarding HPV and p16^INK4a^ status

**Fig 3:**
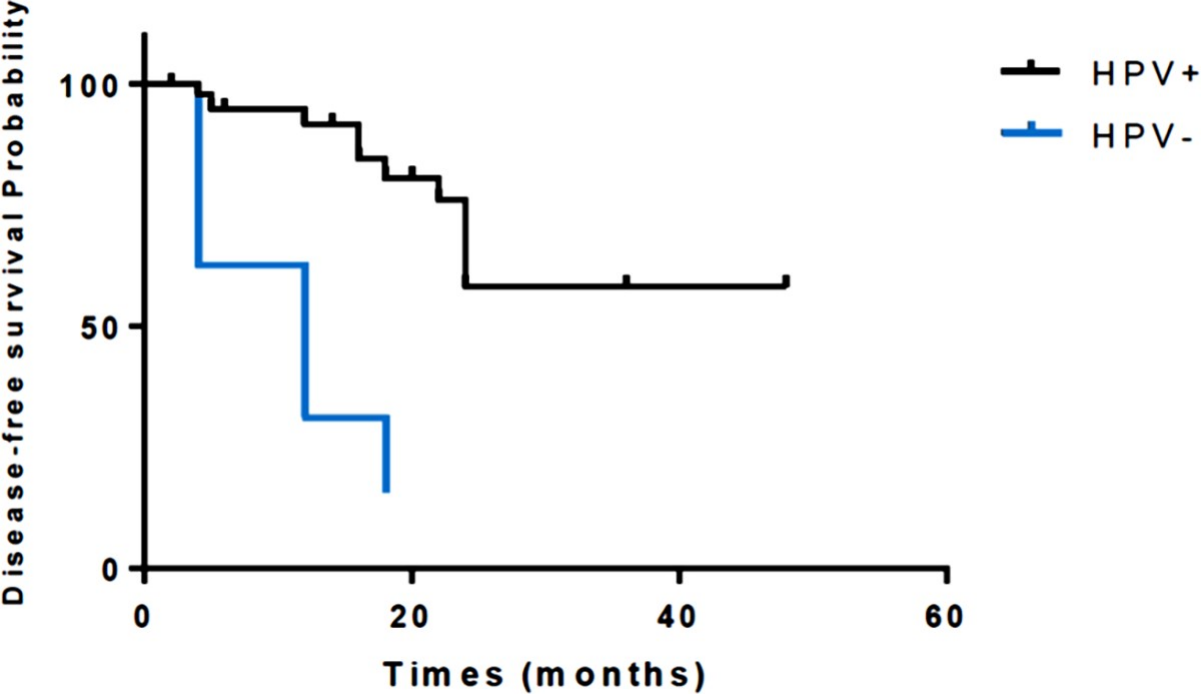
Disease-free survival analysis of HPV+ patients (N = 49) compared to HPV- patients (log-rank p< 0.001).

Regarding the analysis of overall survival and disease-free survival for HPV and p16^INK4a^ status, it was observed that HPV-negative patients had a lower disease-free survival (log rank p< 0.001), whereas in relation to p16^INK4a^ expression, there was no statistical difference in the analysis of overall survival and disease-free survival (Fig 3).

## Discussion

The p16^INK4a^ protein is presently used as an alternative marker for HR-HPV infection in cervical and other carcinomas. Some studies have shown that the overexpression of p16^INK4a^ protein in PC is related to HPV infection [6, 7]. However, other studies did not find such a relationship [29, 30]. Thus, the aim of the present study was to analyze whether expression of p16INK4a is associated with the presence of HPV, histological parameters, and survival in PC. Unlike in previous studies, the PCR technique used in this study was performed using fresh tissue rather than paraffin-embedded tissue, and the immunohistochemistry assays did not use tissue microarray (TMA) sections, which allowed for greater tumor sampling.

In this study, HPV DNA was detected using PCR in 89.1% of cases. This is the highest HPV index ever detected in PC, as data from the literature reveal that only 30 to 50% of cases of PC present evidence of HPV infection [6, 8, 31, 32]. This high index can be explained by the use of fresh and non-paraffin embedded tumor tissue, because according to Iftner and Villa, paraffin-embedded samples are more difficult to analyze owing to greater DNA degradation when compared to fresh samples, in which the DNA is more intact [33]. This finding suggests that the index of cases associated with HPV may be greater than previously described in the literature, highlighting the role of HPV infection in the pathogenesis of PC lesions. HR-HPV was present in 93.9% of these cases, which is consistent with the results of other PC studies [6–8].

Most of the cases in the present study presented with an infection of HPV 16 subtype. The high frequency of this subtype in PC has been reported in previous studies, emphasizing its importance as the most common type of HPV associated with genital cancer [34–36]. In our study, the HPV18 subtype was not observed. It is important to emphasize that in other studies on PC, a low frequency of HPV 18 was found [34, 37].

In the current study, HPV was not found to be associated with any histological prognostic parameters, with the exception of the basal histological tumor subtype, in which all six cases (100%) were found to be high-oncogenic risk HPV. According to a study by Ferrándiz-Pulido et al., the usual HR-HPV-related penile carcinomas seem to have better prognoses compared to the carcinomas related to HR-negative HPV [7]. In our sample of usual carcinomas, 19 (70.4%) had HR-HPV, and there was no evidence regarding an association with histological factors.

HPV-negative patients had a lower disease-free survival compared to HPV-positive patients. This result agrees with the findings of Ferrándiz-Pulido et al. [7], and supports the hypothesis that there are two pathways related to carcinogenesis: one dependent on HPV infection and another independent [35]. HPV-related cancers are believed to have a distinct biology and often show better responses to treatment, making HPV identification critical to treatment efficacy or therapeutic response [38].

As seen in other neoplasms, several histological parameters have been associated with PC prognosis. These include HPV status, histological grade, tumor thickness, lymphatic or venous embolization, tumor subtype, clinical stage, and lymph node involvement [39–42]. In the present study, expression of the p16^INK4a^ protein was only associated with tumor subtype, with p16^INK4a^ overexpression observed in all cases. However, no association was observed with other histological tumor characteristics such as size, clinical stage, histological grade, or lymphatic or perineural invasion. A study of 202 cases of penile carcinomas showed that p16^INK4a^ expression was more likely in tumors with basaloid morphological characteristics [6]. This finding suggested that the expression could be used as a tool in the differential diagnosis of penile carcinoma subtypes [6].

According to Cubilla et al., p16^INK4a^ positivity in PC is strongly related to the presence of the high-risk oncogenic HPV genotype [6, 8]. Therefore, negativity would indicate infection with a low-oncogenic risk genotype or absence of HPV infection [6, 8]. In the present study, a significant association was identified between the expression of p16^INK4a^ and the presence of HR-HPV. Among the LR-HPV cases, one of them was p16^INK4a^ positive. This fact may be explained by the possibility of non-HPV-related oncogenesis involving the pRb pathway, or the capability of some LR-HPV genotypes to induce complete malignant transformation in infected epithelial cells acting as HR-HPV [6]. Furthermore, among the HR-HPV cases, most (25/46) were p16^INK4a^ negative, which may be related to inactivation of the gene encoding p16^INK4a^ (*CDKN2A*), loss of heterozygosity, or promoter hypermethylation. In addition, studies on cervical carcinomas associated with HPV show maximum p16^INK4a^ expression in carcinoma *in situ*, and a reduction in expression as the carcinomas become invasive [6, 43].

As in other studies, some limitations to this research were noted. One of the challenges of this study was to identify which p16^INK4a^ staining pattern should be considered as a cutoff point for p16^INK4a^ positivity. Another limitation was the inability of some patients to undergo follow-up, because most included subjects lived in rural areas far from capital cities and may not have returned for follow-up after the surgery.

## Conclusions

In this study, the expression of p16^INK4a^ protein was found to be associated with the presence of high-risk oncogenic HPV in penile carcinoma samples. Therefore, this protein may serve as an alternative marker for the presence of the virus. Results also showed that the p16^INK4a^ protein is associated with tumor subtype but not with other histological prognostic parameters. Although HPV+ patients exhibited a higher disease-free survival, p16^INK4a^ was not associated with patient survival. In addition, we found the highest incidence of HPV ever recorded in PC, with an absolute predominance of high-risk oncogenic HPV. This finding may be explained by the fact that this study was the first to use fresh tissue samples instead of paraffin-embedded tissues.

## Supporting information

S1 Dataset General Data of all study sets. https://doi.org/10.17605/OSF.IO/3MUV4.
(XLSX)

S1 Protocol Immunohistochemical of p16^INK4a^ protein. https://doi.org/10.17605/OSF.IO/V4SU2.
(DOCX)

S2 Protocol HPV detection and genotyping method. https://doi.org/10.17605/OSF.IO/MHFD9.
(DOCX)

## Abbreviations

PC: Penile cancer
HPV: Human papillomavirus
HR-HPV: High-oncogenic risk HPV
pRb: Retinoblastomaprotein
DNA: Deoxyribonucleicacid
PCR: Polymerase chain reaction
AJCC: American Joint Committee on Cancer
LR-HPV: Low-oncogenic risk HPV
TMA: Tissue microarray
