# Supplementary material for: P16INK4a expression in patients with penile cancer
Source: PLoS One. 2018 Oct 12;13(10):e0205350. doi: 10.1371/journal.pone.0205350 (PMC6185731; doi:10.1371/journal.pone.0205350)
Supplement: S1 Protocol — https://doi.org/10.17605/OSF.IO/V4SU2. (DOCX) [file pone.0205350.s002.docx]

**S1 Protocol. Immunohistochemical of p16^INK4a^ protein**

1. Deparaffinizesections in xylene, 2 to 5 minutes.

2. Hydrate with ethanol absolute, 2 to 3 minutes.

3. Hydrate with 95% ethanol, for 1 minute.

4. Rinse with distilled water.

5. Incubate in alkaline buffer (EDTA pH 8.0) for 1 hour in a pressure cooker (98ºC) for antigen retrieval(masked by formalin fixation and paraffin embedding).

6. Wash withPhospate Buffered Saline(PBS 1x), pH 7.4.

7. Block endogenous peroxidase with3% H_2_O_2_, for 10 minutes.

8. Wash with PBS 1x (pH 7.4).

9. BlockProtein A with bovine serum albumin (1%),for 10 minutes.

10. Incubate with p16^INK4A^ monoclonal primary antibody Clone G175-405 (Zeta Corporation, Arcadia, CA, USA), dilution of 1:75 μL,for1 hour.

11. Wash with PBS 1x (pH 7.4).

12. Incubate with polymer (Spring), for 30 minutes.

13. Wash with PBS 1x(pH 7.4)

14. Incubate with 3,3’ Diaminobenzidine (DAB) chromogendiluited in substrate buffer (1:1000 μL), for 5 minutes.

15. Wash with PBS 1x(pH 7.4).

16. Counterstain with hematoxylin, for 30 seconds.

17. Dehydrate in alcohol/xylene bathsandbladeassembly.

Note:

All incubations are to be performedin a humidified chamber at room temperature.
