# Supplementary material for: P16INK4a expression in patients with penile cancer
Source: PLoS One. 2018 Oct 12;13(10):e0205350. doi: 10.1371/journal.pone.0205350 (PMC6185731; doi:10.1371/journal.pone.0205350)
Supplement: S2 Protocol — https://doi.org/10.17605/OSF.IO/MHFD9. (DOCX) [file pone.0205350.s003.docx]

**S2 PROTOCOL. HPV DETECTION AND GENOTYPING METHOD**

**I - POLYMERASE CHAIN REACTION TECHNIQUE –PCR**

The viral DNA is amplified by *nested* PCR. In the first PCR, PGMY09/PGMY11 primers are used, and in the second,**GP5+/GP6+** primers, according to protocol described below.

**1-PCR PGMY09/PGMY11**

The components of the reaction solution are as follows: 2.5 μL of 10X Buffer; 0.5 μLof MgCl 2 (stock = 50 mM); 0.2 μL of the dNTP mix(stock = 25 mM); 1 μL of the primer mix PGMY09 and 1 μLof PGMY11 primer mix; 0.2 μL of Taq Platinum DNA polymerase (stock =5 U/μL); and approximately 100 ng of genomic DNA. The final mix is 25 μL for each sample. Cycles are as follows: 94°C for 5 minutes, followed by 40 cycles of 94°C for 40 seconds, 55°C for 40 seconds, and 72°C for 40 seconds; and finally 72°C for 5 minutes.

**2- PCR GP5+/GP6+:**

After the first PCR, the second PCR is performed, and the components of the reaction solution are as follows: 2.5 μL of 10X Buffer; 1.5 μL of MgCl 2 (stock = 50 mM); 1.0 μL of the dNTP mix (stock = 25 mM); 2.5 μL of each primer (stock =10 mM); 0.3 μL of Taq platinum DNA polymerase (stock = 5 U/μL); and 2 μL of the PGMY09/PGMY11 PCR product.The final mix is 20 μL for each sample. Cycles are as follows: 94°C for 5 minutes, followed by 40 cycles of 94°C for 40 seconds, 40°C for 40 seconds and 72°C for 40 seconds; and finally 72°C for 3 minutes. Samples are stored at -20°C. The generated product is approximately 110 bp.

**3- ELECTROPHORESIS**

To **confirm the presence of the PCR products**, 5μL of each reaction is run **on 1.5% agarose gels** (at 4 volts/cm) for 40 minutes. As running buffer, 0.5% TBE (0.045M Tris-borate and 0.001M EDTA) is used. Subsequently, the gel is photo-documented under ultraviolet light.

**II – HPV GENOTYPING-SEQUENCING TECHNIQUE**

After visualization of the second PCR product, the amplified DNA is subjected to automatic sequencing using the ABI PRISMTM 310 Big Dye Terminator v3.1 Matrix Standards Kit (Applied Biosystems). The proviral DNA strands are sequenced in a single direction using the primer GP5+, using the ABI PRISM 3500 XL Genetic Analyzer (Applied Biosystems) automatic sequencing equipment.For each reaction, with a final volume of 10 μL, the following reagents are mixed in a previously identified tube: Ultrapure water 10 μL; PCR product 1.0 μL; Primer GP5+ (10 pmol/μL) 0.5 μL; Big Dye0.5 μL; and Syber Safe 3.0 μL.

Subsequently,the tubes containing the reagent mixture are placed in a thermocycler and subjected to a 5 minute cycle at 94°C, followed by 45 cycles of45 seconds at 94°C, 1minute at 60°C, and 1minute at 72°C. At the end of the process, the samples are cooled to 15°C.

**Precipitation of Sequenced DNA**

Sephadex solution (750 μL)is added to columns attached in 2mLmicrotubes and centrifuged for4minutes at 4,400 rpm. Following,the precipitated liquid in the microtube is discarded, and 5 μLof the sequencing product is added in the columns. Subsequently, it is centrifugedfor4minutes at 4,400 rpm. After centrifugation, the column is discarded, and 10μL of formamide is added to the sequencing product remaining in the tube. The resulting solution is applied to the sequencing plate.

**Analysis of Nucleotide Sequences**

All nucleotide sequences obtained are placed in the MEGA 6.0 software. Subsequently, the sequences are compared with those of the GenBank/NCBI database, using the Blast­—Basic Local Alignment Search Tool—as a genotyping tool (<http://blast.ncbi.nlm.nih.gov/Blast.cgi>).
